# Expanding the UniFrac Toolbox

**DOI:** 10.1371/journal.pone.0161196

**Published:** 2016-09-15

**Authors:** Ruth G. Wong, Jia R. Wu, Gregory B. Gloor

**Affiliations:** Department of Biochemistry, University of Western Ontario, London, Ontario, Canada; Wilfrid Laurier University, CANADA

## Abstract

The UniFrac distance metric is often used to separate groups in microbiome analysis, but requires a constant sequencing depth to work properly. Here we demonstrate that unweighted UniFrac is highly sensitive to rarefaction instance and to sequencing depth in uniform data sets with no clear structure or separation between groups. We show that this arises because of subcompositional effects. We introduce information UniFrac and ratio UniFrac, two new weightings that are not as sensitive to rarefaction and allow greater separation of outliers than classic unweighted and weighted UniFrac. With this expansion of the UniFrac toolbox, we hope to empower researchers to extract more varied information from their data.

## Introduction

In 2005, Lozupone et al introduced the UniFrac distance metric, a measure to calculate the difference between microbiome samples that incorporated phylogenetic distance [1]. The goal of the UniFrac distance metric was to enable objective comparison between microbiome samples from different conditions. In 2007, Lozupone added a proportional weighting to the original unweighted method [2]. Since then, papers reporting these metrics have garnered over a thousand citations, and enabled research about everything from how kwashiorkor causes malnutrition [3] to how people can have similar microbiomes to their pet dogs [4]. Except for generalized UniFrac, used to make hybrid unweighted and weighted UniFrac comparisons [5], few advances in the metric have occurred since 2007. In this paper we examine data sets where UniFrac gives misleading results, and present and discuss some alternative weightings for UniFrac.

**Operational Taxonomic Units**

Unlike more distinct species, such as mammalian species, bacterial species are not well defined. Bacterial genomes are highly variable, and regions used to identify bacteria vary in a continuum rather than clusters of similar sequences.

Historically bacteria that have 97% identity in a 16S rRNA gene variable region are considered to be the same taxa [6]. The 97% cutoff was arbitrarily chosen to best map sequence data to bacterial classifications. This threshold is thought to maximizes the grouping of bacteria classified as the same species while minimizing the grouping of bacteria classified as different species [7]. Before sequencing bacterial classification was often done by appearance or by metabolic products, so there are outliers where bacteria classified in the same species are actually genetically very different, or bacteria classified in different genus are genetically very similar.

However, it is difficult to determine how a batch of sequences should be partitioned into groups of 97% identity. One way is to perform a clustering algorithm (using software such as UCLUST [8]) that partitions the groups and then later assign taxonomic identity by matching the seed or central sequences with public databases, such as SILVA [9], the Ribosomal Database Project [10], or Greengenes [11]. Another method is closed reference OTU picking, which starts off with seed sequences from known bacteria and perform the clustering such that the 97% identity groups are centered on the seed sequences. In any case, the resulting taxonomic groupings are known as Operational Taxonomic Units (OTUs), and are used consistently within the same experiment. While OTUs can be annotated with standard taxonomic names such that results can be compared between experiments, technically the taxonomic groupings used by different experiments are not the same, except with closed reference OTUs, or individual sequence unit methods. Individual sequence unit (ISU) methods which do not use OTUs can be run with software such as DADA2 [12].

Grouping of amplicon sequences into OTUs allows for the data to be summarized into a table of counts per OTU per sample.

### 1 Data

UniFrac requires two pieces of information: a phylogenetic tree and a table of counts per inferred taxa per sample. These are derived from a gene tag sequencing experiment, such as the commonly used 16S rRNA gene [13]. The sequenced gene contains a variable region, allowing the sequences to be grouped into OTUs as described in the previous section. A count table can then be generated with the number of reads per OTU per sample. The center sequence of each OTU group can be put into a multiple sequence alignment, from which a phylogenetic tree can be inferred.

The phylogenetic tree is created through a multiple sequence alignment with the representative OTU sequences, using software such as MUSCLE [14] and FastTree [15], or using a guide tree, such as through Greengenes [11] or the QIIME software [16]. Each leaf of the tree represents one of the OTUs, and each of the branches of the tree has a length. Additionally, the tree needs to be rooted for the UniFrac calculation to be performed. This is often done by rooting the tree at its midpoint.

### 2 Compositional Data Analysis

Microbiome data is in the form of a list of counts per feature (OTUs in this case), with the features composing an aspect of the microbiome for each sample. This is compositional data because the total sum of reads for a sample is arbitrary, being determined by the capacity of the sequencing instrument [17] [18] [19]. There are several core truths about microbiome data and its compositional nature that should be considered when making an analysis strategy.

First, the total number of reads per sample is influenced by sample collection, extraction, sequencing library preparation, and sequencing platform, and is irrelevant to the biological implications of the data. Additionally, the constraint of the count total causes the abundance of different taxa to appear to be negatively correlated with each other when analyzed by conventional statistics [20]. When one taxa increases in abundance, the counts detected in other taxa decrease in abundance, even if the taxa are not decreasing in abundance biologically. For example, one study compared the microbiome of vaginal swab samples from women with bacterial vaginosis (BV), women without BV, and women with intermediate BV, using qPCR to quantify the taxa [21]. *Prevotella* was found to increase through non-BV to intermediate to BV, while *Lactobacillus iners* stayed relatively the same [21]. If the same samples were put through a gene tag sequencing experiment where the taxa could not be quantified and the total read counts were constrained, one might incorrectly conclude that the abundance of *Lactobacillus iners* was decreasing while *Prevotella* was increasing.

To prevent incorrect conclusions, data should be analyzed in a compositional way. In Euclidean space, data points can increase or decrease freely. Compositional data is under a sum constraint, and exist in a non-Euclidean space known as the Aitchison simplex [22]. A data transformation can be performed to put the data into Euclidean space, so that it can be analyzed with standard statistical methods that depend on Cartesian coordinates and linear relationships. These transformations involve examining the ratios of different OTU abundances to each other, so that the total number of reads do not unduly affect the result [23] [24]. In the example with bacterial vaginosis, using ratios of taxa to each other would elucidate the nature of the biological change in the data.

### 3 Unweighted UniFrac

**Fig 1:**
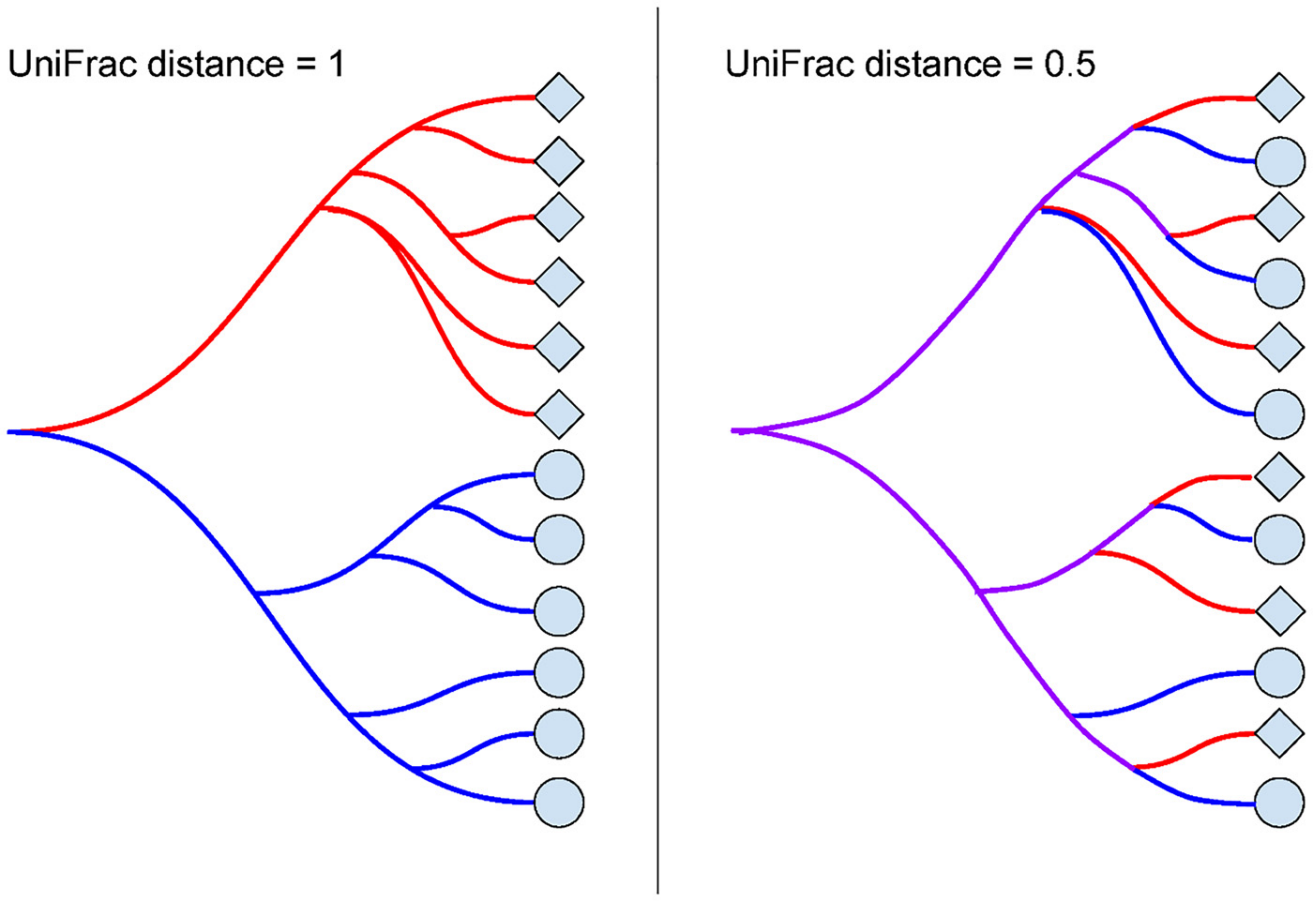
Unweighted UniFrac. When two samples do not share any branches of the phylogenetic tree, the unweighted UniFrac distance is maximized at 1. When two samples share half of their branch lengths on the phylogenetic tree, the unweighted UniFrac distance is 0.5. If the two samples contain exactly the same taxa, the unweighted UniFrac distance is minimized at 0, since the samples share all branches.

Unweighted UniFrac [1] uses an inferred evolutionary distance to measure similarity between samples. It requires a reference phylogenetic tree containing all the taxa present in the samples to be examined, plus information about which taxa were detected in each sample. The calculation is performed by dividing the branch lengths that are not shared between the two samples by the branch lengths covered by either sample. Fig 1 shows example calculations for UniFrac based on the tree overlap. A distance of 0 means that the samples are identical, and a distance of 1 means that the two samples share no taxa in common.

As UniFrac is a binary test of absence, it is sensitive to sequencing depth, and assumes that the data has been normalized to a common sequencing depth [25]. Thus, rarefaction prior to unweighted UniFrac has become a standard part of the microbiome analysis workflow, with built in rarefaction functions in QIIME [16] and mothur [26].

### 4 Weighted UniFrac

Weighted UniFrac [2] is an implementation of the Kantorovich–Rubinstein distance in mathematics, also known as the earth mover’s distance [27]. Rather than looking only at the presence or absence of taxa, each branch length of the phylogenetic tree is weighted by the difference in proportional abundance of the taxa between the two samples.

This technique reduces the problem of low abundance taxa being represented as a 0 or by a low count depending on sampling depth. In unweighted UniFrac, such taxa would flip from absent to present, and could skew the measurement: this would be especially problematic if the taxa are on a long branch. In weighted UniFrac, low abundance taxa have a much lower weight and so will have a lower impact on the total distance reported by the metric.

UniFrac is constituted as either a binary weighting (unweighted UniFrac) [1], a linear proportion (weighted UniFrac) [2], or some combination of the two (generalized UniFrac) [5]. However, it is a misconception that the data are linear because the sum of the total number of reads is constrained by the sequencing machinery [28] [17] [18] [20] as described above.

Microbiome communities can exhibit tremendous variation in their total bacterial count. For example, a stool sample may produce more highly concentrated DNA extract than a skin swab sample, resulting in a different number of input molecules but a similar read count total. Vaginal samples from patients with bacterial vaginosis compared to patients without can have DNA extract concentrations that differ one magnitude [21]. Alternative weightings and non-linear transformations of data need to be explored. Furthermore, unweighted UniFrac is known to be unreliable, but it is not generally understood how this can impact results.

## Materials and Methods

### 5 Analytical techniques

#### Rarefaction

Rarefaction normalizes the samples OTU counts to a standard sequencing depth by sampling without replacement [29]. This resulting table can be thought of as a random point estimate of the dataset, as the output is a sub-sample without replacement of the original table. This standardization process is recommended by the authors of UniFrac [30] in order to account for the sensitivity of UniFrac to sequencing depth.

Rarefactions can be performed using the QIIME software [16] or using the vegan package in R [31].

#### Unweighted UniFrac

Unweighted UniFrac is calculated based on the presence or absence of counts for each branch in the phylogenetic tree, when comparing two samples. A branch belongs to a sample when at least one of the OTUs in the leaves below it have a non-zero abundance. The formula for unweighted UniFrac is as follows, where *b* is the set of branch lengths in the phylogenetic tree, *A* and *B* represent the two samples being compared, △ is the symmetric difference between two sets, and ∪ is the union between two sets:

$$Unweighted_{AB}=\frac{\sum_{}b_{A}\Delta b_{B}}{\sum_{}b_{A}\cup b_{B}}$$

The sum of the branch lengths that belong to one sample but not the other is divided by the sum of the branch lengths that belong to one or both samples.

**Fig 2:**
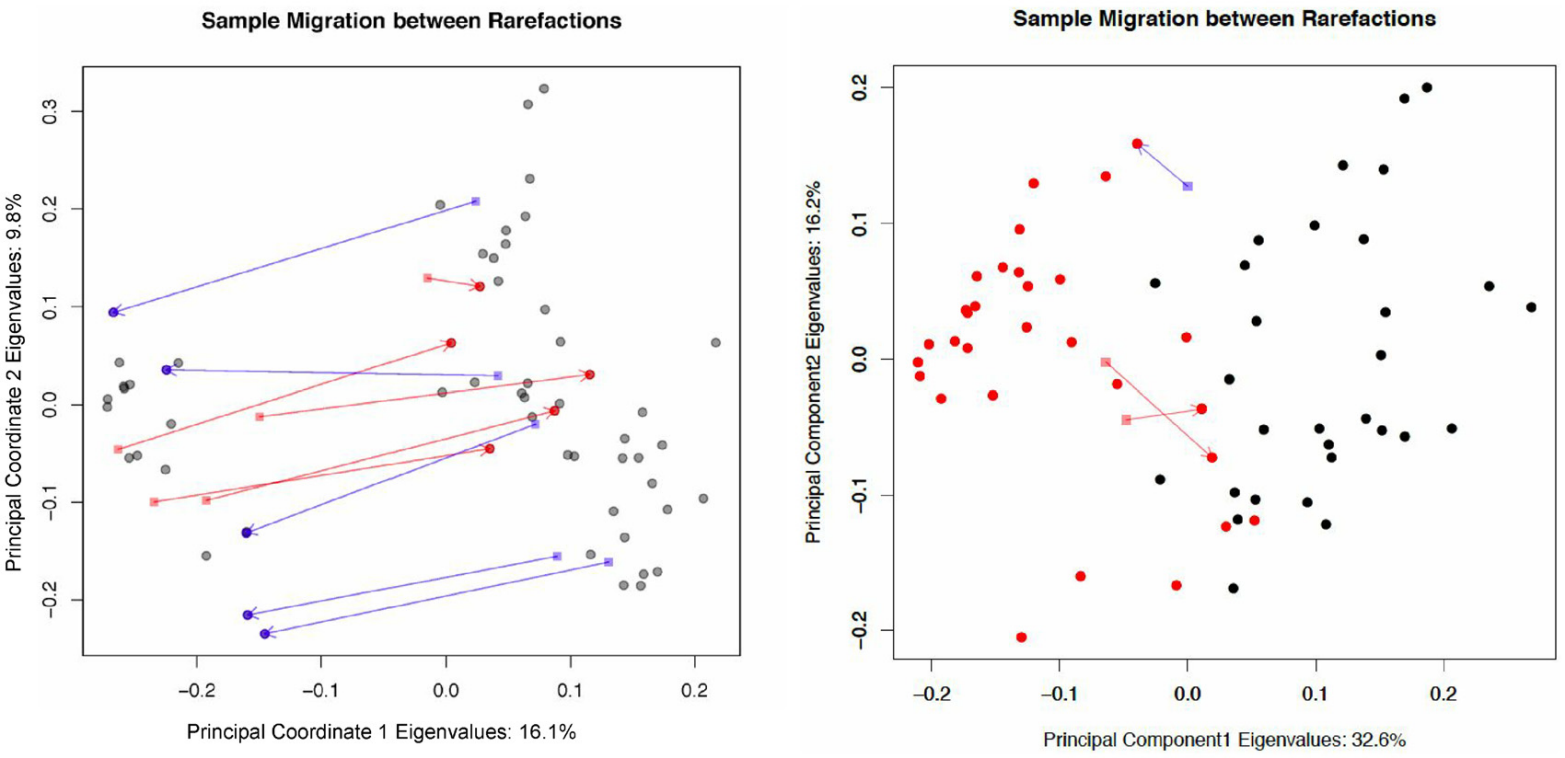
Sample migration in different rarefactions, plotted on principal coordinates, measured with unweighted UniFrac. The left plot is of the tongue data set while the right plot is the tongue dorsum vs. buccal mucosa data set. On the left panel red samples have moved from the left cluster to the right cluster between rarefactions. Blue samples have moved from the right cluster to the left. Samples are taken from the tongue dorsum body site from the Human Microbiome Project database. If the experiment were run once, one might mistakenly assume that there are two clusters of data, however, the inconsistent sample membership of the two groups between rarefactions proves the clustering irreproducible. The tongue dorsum and buccal mucosa data set is included for comparison, with the tongue samples colored black and the buccal mucosa samples colored red. Note that the variance explained in the tongue data set by the first and second coordinate is merely 16.1% and 9.8% respectively, indicating that the data is rather spherical, even though the points on the plot appear to show two separated clusters (compare with 32.6% and 16.2% in the tongue dorsum vs. buccal mucosa data set). The variance explained in the first and second coordinate in the 2011 UniFrac commentary [25] was even smaller, at 8.6% and 5.6%.

Note that the implementation of unweighted UniFrac in QIIME (see Fig 2) and also GUniFrac (see S1, S2 and S3 Figs) includes a tree pruning procedure, where the tree is pruned to only include OTUs that are present in each pairwise sample comparison. Except for in Fig 2 and S1, S2 and S3 Figs, the scripts used in this paper do not prune the tree, in order to be consistent with weighted UniFrac. In weighted UniFrac, pruning the tree makes the measurement a dissimilarity rather than a distance (S4 Fig).

#### Weighted UniFrac

Weighted UniFrac [2] also incorporates each branch length of the phylogenetic tree, and weights them according to proportional abundance of the two samples. The formula for weighed UniFrac is as follows, where *A* and *B* are the two samples, *b* is the set of branch lengths, and $\frac{A_{i}}{A_{T}}$ and $\frac{B_{i}}{B_{T}}$ are the proportional abundances associated with branch length *b*_*i*_:

$$Weighted_{AB}=\frac{\sum_{i}^{n}\left(b_{i}\;\times \;\left|\frac{A_{i}}{A_{T}}-\frac{B_{i}}{B_{T}}\right|\right)}{\sum_{i}^{n}b_{i}}$$

#### Information UniFrac

Information UniFrac is calculated by weighing each branch length by the difference in the uncertainty of the taxa abundance between the two samples. Uncertainty or information (*I*) is calculated as follows, where p is the proportional abundance [32]:

$$I\;=\;-p\;\times \;log_{2}\left(p\right)$$ (1)

If a sample is composed of 50% taxa A and 50% taxa B, then the proportional abundances have maximum uncertainty about what taxa is likely to be seen in a given sequence read. If a sample is 80% taxa A and 20% taxa B, then there is less uncertainty about both taxa, because a given sequence read is more likely to be taxa A and less likely to be taxa B. When the amount of uncertainty that a taxa has in one sample corresponds with the amount of uncertainty the same taxa has in a different sample, the abundance of that taxa is mutually informative between samples. Weighting UniFrac by uncertainty combines the the concept of uncertainty with phylogenetic relationships to identify taxa that are differentially informative between groups.

The formula for Information UniFrac is as follows:

$$Information_{AB}\;=\;\frac{\sum_{i}^{n}\left(b_{i}\;\times \;\left|\frac{A_{i}}{A_{T}}log\left(\frac{A_{i}}{A_{T}}\right)-\frac{B_{i}}{B_{T}}log\left(\frac{B_{i}}{B_{T}}\right)\right|\right)}{\sum_{i}^{n}b_{i}}$$

Information UniFrac approaches a minimum of zero for OTUs in a sample that are at either abundance extreme. It is also related to the Aitchison distance in compositional data analysis [33].

#### Ratio UniFrac

In complex microbiome communities, there may be a large number of bacterial taxa with few counts, such that the data is sparse. Taking the geometric mean of the proportional abundances of taxa in a microbiome sample represents an unbiased baseline of the average abundance of features with geometric growth characteristics—such as bacteria which divide by fission [22]. Experiments generally do not have power to detect differences at abundances below the mean [17]. Centering the proportional abundances around the geometric mean thus allows one to examine the data in this context, muting differences that are close to the baseline abundance and accentuating OTUs that are much more abundant than the mean. The formula for ratio UniFrac is as follows, where *gm* is the geometric mean:

$$Ratio_{AB}\;=\;\frac{\left(\sum_{i}^{n}b_{i}\;\times \;\left|\frac{\frac{A_{i}}{A_{T}}}{gm(A_{i})}-\frac{\frac{B_{i}}{B_{T}}}{gm(B_{i})}\right|\right)}{\sum_{i}^{n}b_{i}}$$

Note that the geometric mean is calculated by combining all children in the subtree of *b*_*i*_ into $\frac{A_{i}}{A_{T}}$ for sample *A* or $\frac{B_{i}}{B_{T}}$ for sample *B*, and including the rest of the single taxa proportional abundances separately. The one combined proportional abundance and the remaining single taxa proportional abundances are input into the geometric mean formula, as set *a*:

$$gm\left(a\right)={\left(\prod_{i}^{n}a_{i}\right)}^{1/n}$$

One challenge when it comes to the analysis of read count data is that the data is very sparse. Whether a low-abundance taxa or feature appears in the data as a zero or a low positive count is up to chance, and assuming that a zero count represents the absence of a taxa can be very misleading [17]. A Bayesian approach can be used to give a posterior estimate of the likelihood for zero count OTUs: this is implemented by the cmultRepl command in the zCompositions package in R [34].

The use of ratio weighting for UniFrac produces measurements that violate the metric triangle inequality, such that Euclidean statistics are technically invalid. Thus this metric, like the Bray-Curtis metric, is a dissimilarity, not a distance.

For this paper, we calculate UniFrac metrics using a custom R script, which includes unweighted UniFrac, weighted UniFrac, information UniFrac, and ratio UniFrac [35]:

#### Bray-Curtis dissimilarity metric

The Bray Curtis dissimilarity metric [36] quantifies how dissimilar two sites are based on counts. A Bray-Curtis index of 0 means that two samples are identical, while a Bray-Curtis index of 1 means samples do not share any species. It is computed as a proportion through the formula:

$$\begin{array}{lll} & C_{ij} & =\;1-\frac{2C_{ij}}{S_{i}\;+\;S_{j}}\\ \text{where} & C_{ij} & =\text{ dissimilarity index bound by [0,1]}\\ & S_{i} & \;=\text{ Specimen counts at site i}\\ & S_{j} & \;=\text{ Specimen counts at site j } \end{array}$$

### 6 Data preparation

The data used comes in the form of a table of counts per operational taxonomic unit per sample, plus a phylogenetic tree. All of our data are derived from 16S rRNA gene tag sequencing experiments, and the data and scripts can be accessed at https://github.com/ruthgrace/r_scripts [37].

#### Tongue dorsum data set

The tongue dorsum data set is a collection of 60 microbiome samples taken from the tongues of healthy participants. There were 0.3 million reads across 554 OTUs, and a minimum and maximum of 659 and 17176 reads per sample.

Samples from this experiment were sourced from the Human Microbiome Project [38] Qiime Community profiling v35 OTU tables (http://hmpdacc.org/HMQCP/).

Rarefaction was conducted through Qiime version 1.8.0-20140103 to 659 reads (the lowest number of reads for a sample), and generation of the ellipse figures was done in R version 3.2.3 (2015-12-10) “Wooden Christmas-Tree” x86_64-apple-darwin13.4.0 (64 bit).

A principal coordinate analysis is drawn from each distance matrix per metric, and for the first principal coordinate of each metric, the resultant value (*V*_*res*_) is computed per each first principal coordinate as defined by the formula:

$$\begin{array}{lll} & V_{res} & =\;\frac{\left|V_{1}-V_{i}\right|}{range\left(V_{1},V_{i}\right)}\\ \text{where} & V_{res} & =\text{ Set of computed PC1s,}\\ & V_{1} & =\text{ Reference PC1 (the first),}\\ & V_{i} & =\text{ Each subsequent PC1,} \end{array}$$

#### Tongue dorsum and buccal mucosa data set

The tongue dorsum and buccal mucosa data set is a collection of 30 microbiome samples taken from the tongues of healthy participants, plus 30 microbiome samples taken from the buccal mucosa (cheek) of a different set of healthy participants. There were 0.4 million reads across 12701 OTUs, and a minimum and maximum of 5028 and 9861 reads per sample. Note that if the OTUs that are less than 1% abundant in all samples are filtered out, only 179 OTUs remain.

To create this data set, thirty random samples were selected from the tongue site of the Human Microbiome Project [38] and thirty random samples from the buccal mucosa site. Samples were filtered so that only samples with 5000 to 10,000 reads were included.

Read counts from the HMP data set were rarefied to the smallest total read count per sample using the vegan R package [31] before the unweighted UniFrac distance was calculated. Weighted, information, and ratio UniFrac were calculated on the data set without rarefaction. The resulting distances were plotted for principal coordinate analysis.

#### Breast milk data set

The breast milk data set is a collection of 58 microbiome samples taken from lactating Caucasian Canadian women. The breast milk data set used here has also been published in a recent study [39]. There were a total of 5.3 million reads across 115 OTUs, and a minimum and maximum of 3072 and 2.8 million reads per sample. Note that the 2.8 million reads came from a sample that was taken from a patient with an infection, and the next largest number of reads per sample was 282485 (ten times less).

The count table was analyzed using our custom UniFrac script, which can be accessed at https://github.com/ruthgrace/ruth_unifrac_workshop [35]. Data was rarefied to the sample with the smallest number of read counts (3072) before the unweighted UniFrac distance matrix was calculated. Non-rarefied data was used for weighted, information, and ratio UniFrac. Data was plotted using a principal coordinates or component plot as appropriate.

#### Monoculture data set

The monoculture data set is simulated based on the infected sample from the breast milk data set. Each simulated sample has exactly the same counts per taxa as the infected sample, except that the taxa are shuffled. After taxa shuffling, the data was manipulated into two groups. In one set of 20 samples the taxa with the highest count was swapped with *Pasteurella*, in another set of 20 the taxa with the highest count was swapped with *Staphylococcus*, and in the last set of 20 the taxa with the highest count was swapped with *Pseudomonas*. These three taxa were picked because they were the most highly abundant in the original breast milk data set. This process produced three sets of monocultures, dominated by the three different taxa.

## Results

### 7 Unweighted Unifrac is highly sensitive to rarefaction instance

A commentary by Lozupone et al. 2011 [25] addressed the sensitivity of Unweighted UniFrac to sampling. Lozupone’s group used mean UniFrac values to compute a confidence ellipse between the first and third quartile. However, we observed that this approach under-represented the true variability of unweighted UniFrac as a distance metric by highlighting how individual samples vary. In the absence of true differences and in the presence of uneven sampling, unweighted UniFrac can be sensitive to rarefaction instances. We show this by analyzing two rarefactions of the same body site with the rationale that if there is no true difference in the data, separation of these samples should not be observed.

Sixty tongue dorsum subsamples were drawn from the Human Microbiome Project data without replacement. Rare OTUs with less than 100 total counts across all the samples were removed. The minimum sample count for the subset of 60 we analyzed was 659, therefore we rarefied (subsampled) to the minimum of 659 to normalize the samples, prior to performing a principal coordinates analysis (PCoA). For Fig 2, two independent rarefactions of the data were conducted in order to observe the effect of rarefaction instance on the metric. The unweighted UniFrac distance was computed for each rarefaction, and Procrustes adjustment was applied in order to overlay the PCoA-derived second rarefaction onto the first. A PCoA of rarefaction 1 was plotted, and any samples that changed between rarefactions one and two were visualized with red and blue on the plot. If the sample moved from one side of the first coordinate axis to the other between the rarefaction instances, it was indicated with either a blue or a red arrow.

In both rarefactions on Fig 2, samples separated distinctly into two clusters on principal coordinate 1. Principal coordinate 1 explains the most variation in the data, and is thus useful to visualize if any associated metadata is behind the sample separation. However, the separation was not explainable by any metadata associated with the HMP experiment, and is thus an undesirable result. When plotting the rarefactions against each other, several samples are observed to be unstable, exhibiting large differences in location. This example demonstrates that samples with little difference can appear to be different through the unweighted UniFrac distance metric and that rarefaction can lead to misleading and non-reproducible results.

**Fig 3:**
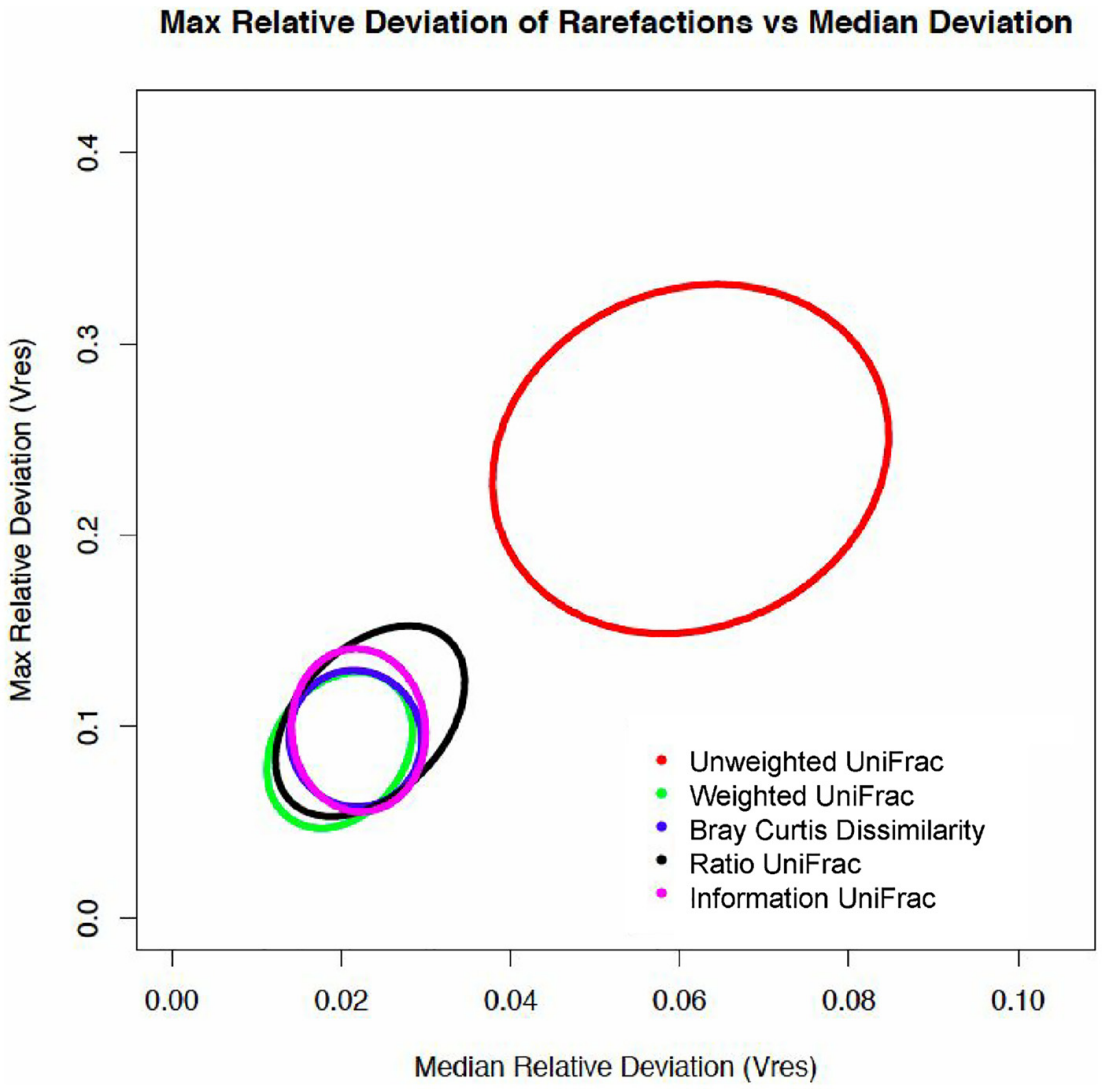
Maxiumum relative deviation of rarefactions versus median deviation for traditional and non-traditional microbiome dissimilarity metrics. Sixty samples from the tongue dorsum were taken from the Human Microbiome Project [38], and rarefied 100 times. The maximum relative deviation was plotted against the median relative deviation of the rarefied data, and ellipses were drawn at the 95% confidence interval, around the cloud of points for each metric. A higher maximum and median devation indicates lower reproducibility of results between rarefaction instances. Both the maximum relative deviation of rarefied data and the median relative deviation of rarefied data are greater in unweighted UniFrac than in weighted UniFrac, Bray Curtis dissimilarity, ratio UniFrac, and information UniFrac.

For the ellipse plot in Fig 3, 60 tongue dorsum subsamples were randomly drawn without replacement. Rare OTUs with less than 100 total counts across all samples were removed. A hundred separate rarefactions were conducted on the data to a minimum sampling depth of 659. For each individual rarefied OTU table, a distance matrix was computed using one of unweighted Unifrac, weighted UniFrac, Bray-Curtis Dissimilarity, information UniFrac, or ratio UniFrac as the weighting method. By generating 100 separate datasets for each metric, it is possible to assess the effect of rarefaction instance on each metric by analyzing what is essentially the same data. In other words, what does the effect of random sampling (rarefaction) have on the output of each metric? Each distance matrix generated per metric was adjusted with a Procrustes adjustment to overlay the subsequent rarefactions onto the first.

The maximum value of Vres for each rarefaction is plotted against the median value per rarefaction in Fig 3. This plotting serves to highlight the maximum potential change for an analysis given that there is no difference in the data. Unweighted UniFrac shows by far the highest maximum potential change between rarefactions, compared to weighted, information, and ratio UniFrac, as well as Bray-Curtis.

Given the wide use of unweighted UniFrac in the literature with small principal coordinate 1 and 2 effects, we suggest caution in their interpretation. For example, see the use of unweighted UniFrac in these papers about the human microbiome published in Cell [40], where the first and second principal coordinates axis explain 14% and 9.5% of the variation in Fig 2A, as well as in Nature [41], where the first principal coordinate explains 14% of the variation in Fig 1. In both of these examples, less variance is explained by the first principal coordinate than in our uniform tongue data set.

### 8 The cause of rarefaction variation by Unweighted Unifrac

One point to note is that rarefaction carries the assumption that microbiota within samples are homogeneous and randomly distributed. However, this assumption is only valid if proper sampling protocols are observed [42]. A combination of unevenly sampled OTUs and distantly related OTUs will contribute to the variability in unweighted UniFrac when OTUs are ultimately rarefied. Distance matrices between samples will be affected when rare OTUs are left out during the rarefaction processes. It becomes intuitive to see how similar samples may grow dissimilar from each other through unweighted UniFrac on rarefied samples as the number of unshared branches increases as OTUs are removed.

**Fig 4:**
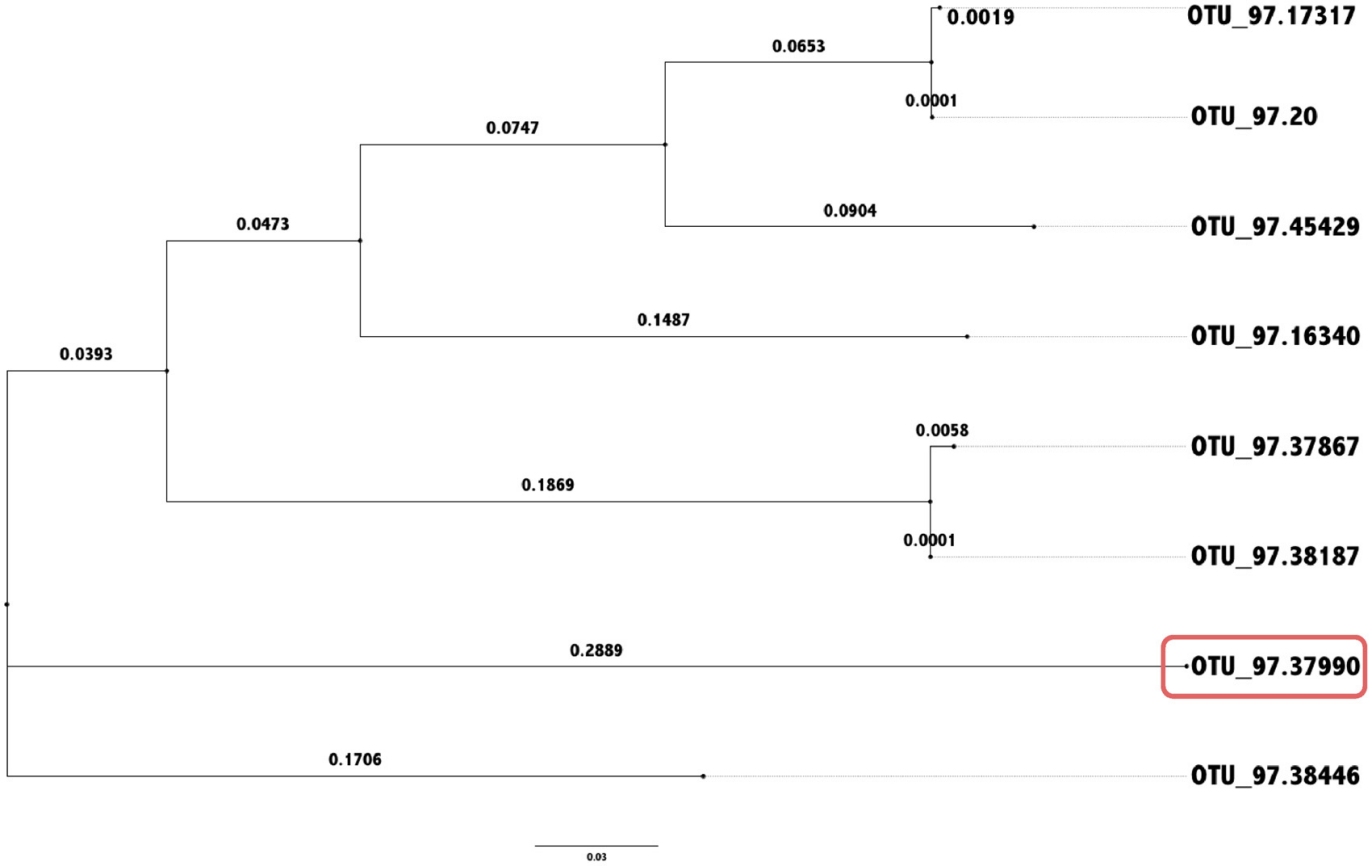
Phylogenetic tree with long isolated branches. Variation in different rarefactions of data in unweighted UniFrac analysis is exacerbated by the presence of long isolated branches in the phylogenetic tree, such as the circled OTU in this example.

With rare OTUs and long branch lengths in the phylogenetic tree (Fig 4), the Unweighted UniFrac distance metric on rarefied data is highly variable, declaring the samples A and B identical (distance of 0) with 1 rarefaction, and different with another (distance of 0.4175), as demonstrated in Table 1 and the calculations above.

**Table 1:** Original abundance of taxa and rarefied abundance of taxa. This data was simulated to demonstrate how rarefaction can change the distances reported by the unweighted UniFrac metric. Originally, sample A contained 1075 counts and sample B contained 221 counts in total. Both samples were rarefied to 221 counts, twice. The OTU in bold has been rarified to a zero count in sample A for one instance and a non zero count in the other instance. In Rarefaction 1, the unweighted UniFrac distance (unshared over total branches) is 0.4175, while in Rarefaction 2 the distance is 1.12.

OTU.ID	A	B	A R1	B R1	A R2	B R2
OTU.16340	52	1	8	1	12	1
OTU.17317	17	4	3	4	5	4
OTU.20	70	18	14	18	20	18
OTU.37867	59	10	9	10	11	10
**OTU.37990**	7	59	**0**	59	**1**	59
OTU.38187	646	115	132	115	122	115
OTU.38446	6	8	0	8	1	8
OTU.45429	218	6	55	6	49	6

While an improvement on unweighted UniFrac, weighted UniFrac can overweight differences between large proportional abundances and underweight differences between small proportional abundances. If one bacterial taxa increased in proportion from 5/1000 to 10/1000 and another taxa increased in proportion from 95/1000 to 100/1000, they would have the same weight in weighted UniFrac. However, the first taxa has doubled in proportion between samples, and this is much more biologically significant than the change in proportional abundance in the second taxa. Additionally, it does not account for how the counts add up to a constrained sum determined by the sequencing machine model. Because the sum is constrained, as with the bacterial vaginosis sample earlier, an increase in growth of one taxa can make the data look like there is a decrease in abundance in other taxa, even if in reality the population of the other taxa stayed the same.

Here we explore some alternatives to unweighted and weighted UniFrac, and discuss their merits and shortfalls.

### 9 Information UniFrac

The difference in information content between taxa with low proportional abundances (which make up the bulk of microbiome data) is generally higher than the difference between the proportional abundances themselves, potentially allowing scientists to differentiate samples with subtle differences when the differences are primarily in low count taxa.

**Fig 5:**
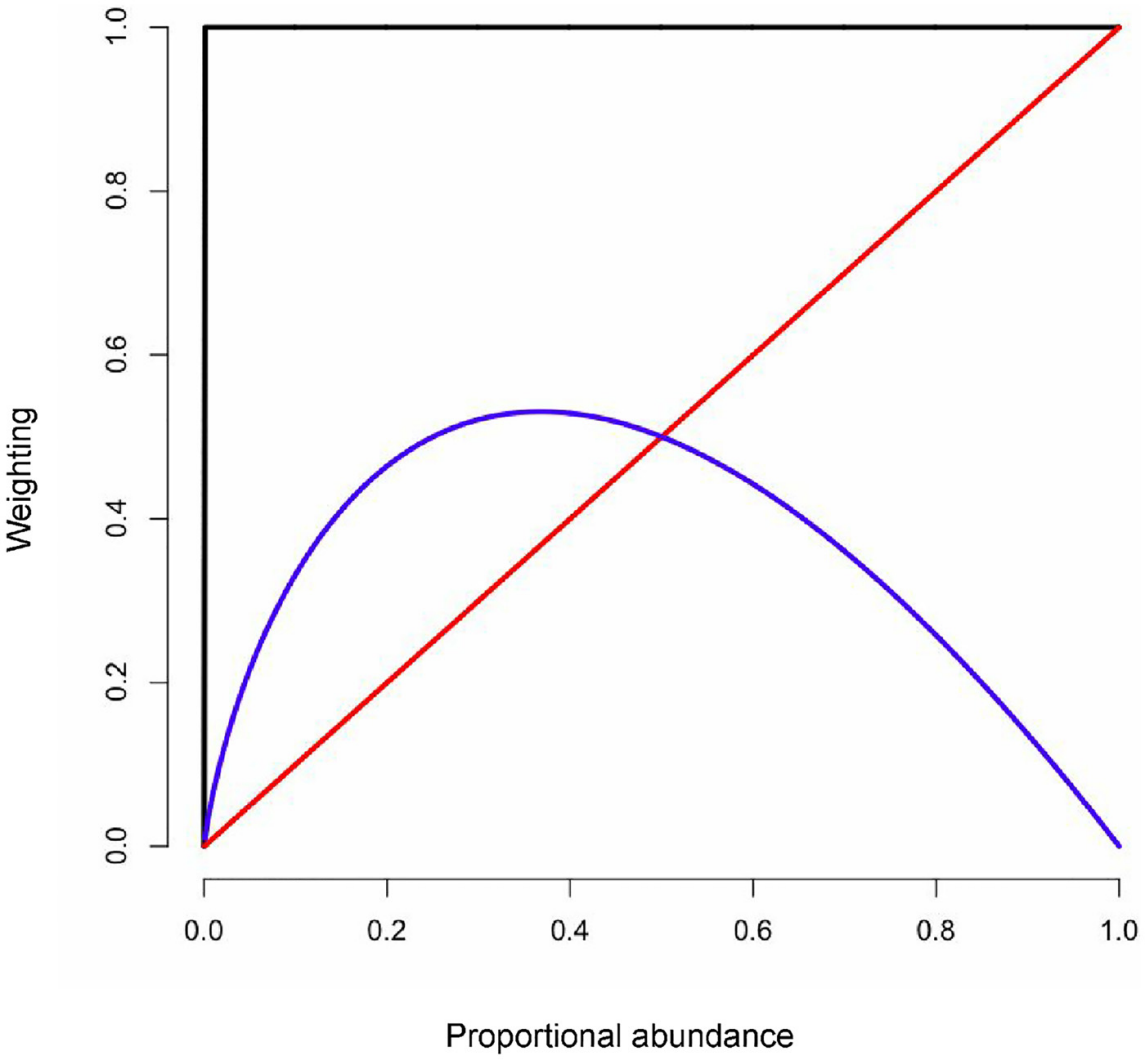
UniFrac weights. Each UniFrac weighting is plotted with the corresponding proportional abundance. The black line is unweighted UniFrac, the red line is weighted UniFrac,and the blue line is information UniFrac. From 0 to 0.2 on the x-axis information UniFrac has a higher slope, and therefore more discovery power for smaller changes in abundance. As the x-axis approaches 1, changes in abundance add little discovery power to information Unifrac.

For example, Fig 5 shows the weighting of a taxon in unweighted, weighted, and information UniFrac as a function of the taxon proportional abundance. Near the 0, 0 point the proportional abundances are low and information is 0. However, small increases in abundance result in large changes in contribution to UniFrac weighting, as shown by the slope of the curve. Here there is higher differentiation between weights of different pairs of low proportional abundances for information UniFrac, as shown by the higher slope of the curved graph. The ratio UniFrac (not depicted) depends on the geometric mean of the taxonomic abundances, and each sample would have a different slope in the weight graph depending on how evenly the abundances were distributed.

### 10 Tongue and buccal mucosa comparison

We next explore two other datasets, one with a defined difference between groups (tongue dorsum compared to buccal mucosa), and one with an outlier that is only apparent when analyzed by certain dissimilarity metrics.

**Fig 6:**
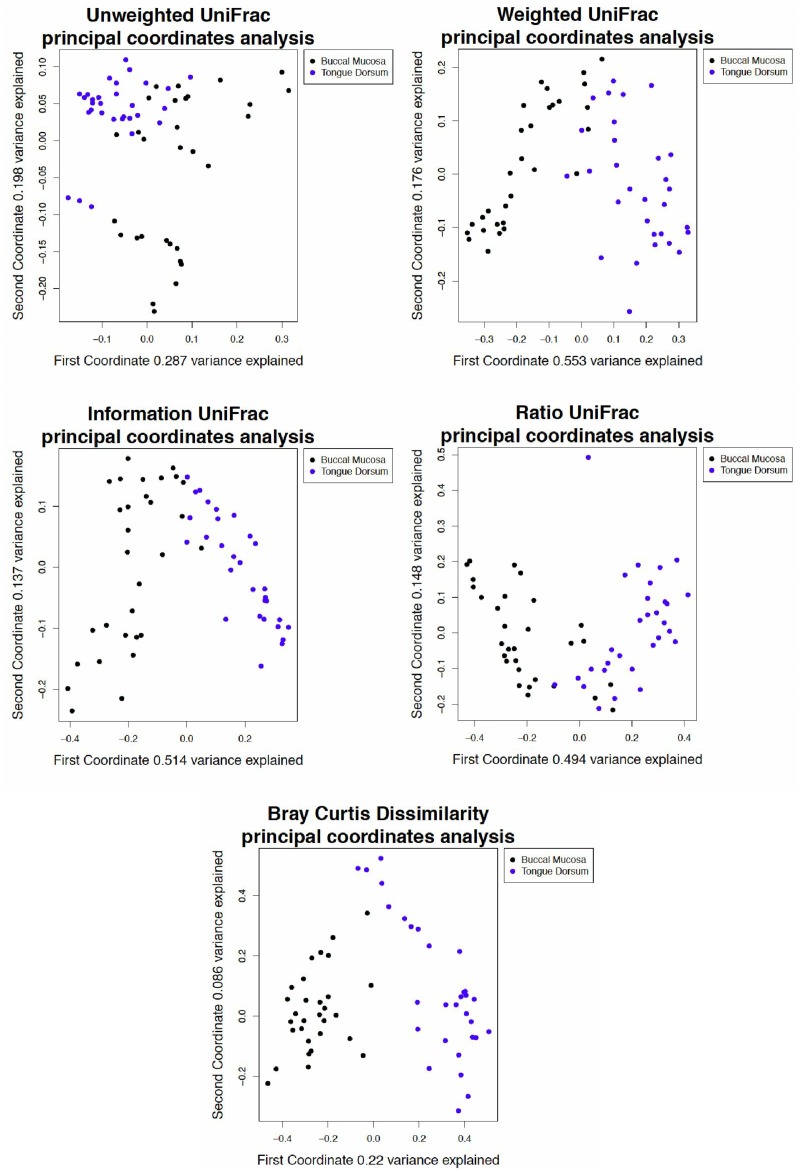
Analysis of tongue and buccal mucosa data using different UniFrac weightings. A principal coordinate analysis of a 16S rRNA gene tag experiment done on samples from the tongue and buccal mucosa, selected from the Human Microbiome Project [38]. All weightings and the Bray-Curtis dissimilarity show separation between the samples by body site. Note that the variance explained by the first and second principal coordinate axis is higher than in the tongue-tongue data set from Fig 2, which had 16.1% and 9.8% variance explained, respectively.

Fig 6 shows a principal coordinate analysis plot with four different metrics: unweighted UniFrac, weighted UniFrac, information UniFrac, and ratio UniFrac. We observe that the difference in the microbiome between the human tongue and buccal mucosa are well defined by all metrics (Fig 6), since all of the weightings show separation between the samples according to body site. We conclude from (Fig 3) that weighted UniFrac, information UniFrac, and ratio UniFrac do not tend to show spurious separation in uniform data sets to the degree that unweighted UniFrac does, while reliably separating samples in data with a defined difference between groups.

### 11 Breast milk Data

**Fig 7:**
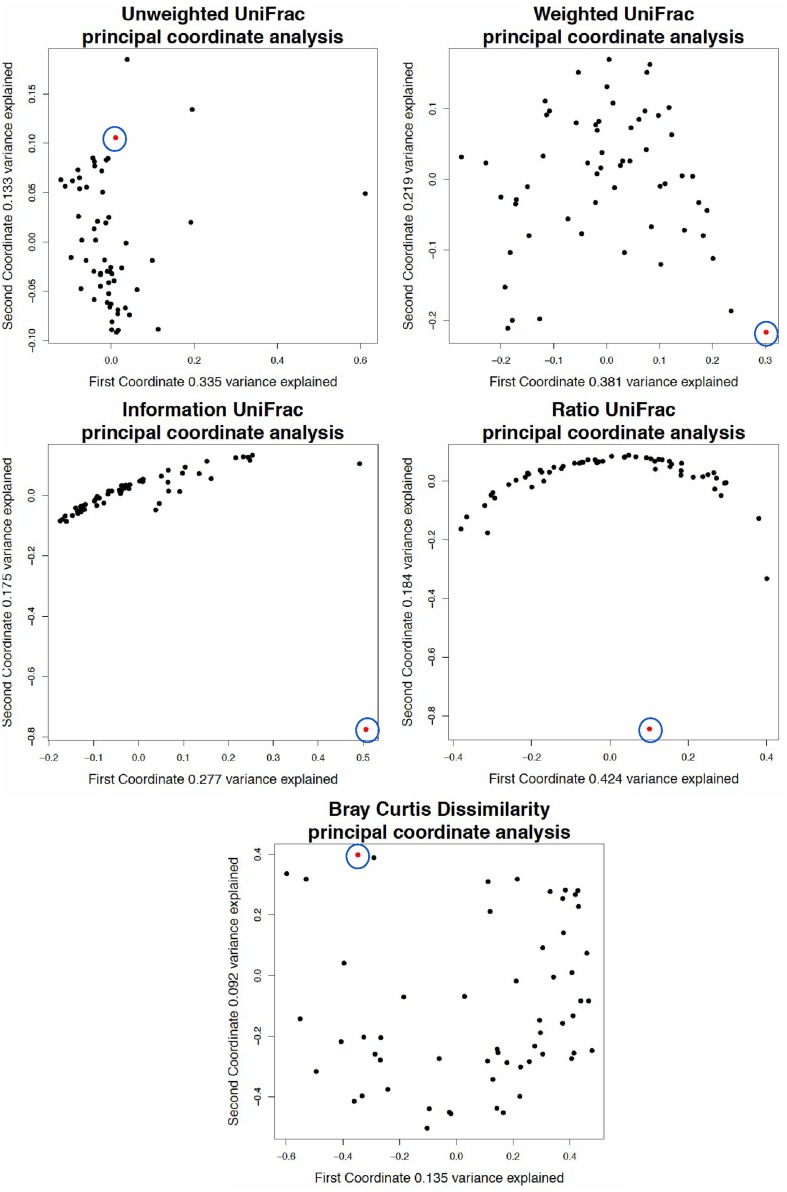
Analysis of breast milk data using different UniFrac weightings. A principal coordinate analysis of a simulated 16S rRNA gene tag experiment based on the breast milk data. Red samples are dominated at 07% by *Pasteurella*, black samples are dominated by *Staphylococcus*, and cyan samples are dominated by *Pseudomonas*. Note that while information Unifrac appears to separate the samples reasonably well visually, the amount of variance explained by the first two coordinates is much lower than even weighted UniFrac.

Fig 7 is a principal coordinate analysis of a 16S rRNA gene sequencing experiment done on microbiome samples from breast milk [39]. Breast milk samples were collected and the V4 region of the 16S rRNA gene was sequenced. One of the patients who provided a sample had an active infection, producing a sample that consisted of 97% *Pasteurella*. We noted that this sample was not distinct in unweighted and weighted UniFrac because the distance from the *Pasteurella* branches of the phylogenetic tree to the root of the tree (rooted by midpoint) were not particularly short or long, measuring at just over the 3rd quartile of all root-to-leaf distances. In addition, the *Pasteurella* leaves shared a clade with many other taxa.

The reason the infected sample in the breast milk study is so distinct from the rest of the samples in Information UniFrac and Ratio UniFrac is because of the weighting. The infected sample was 97% *Pasteurella*, while the other samples generally had 15-20% each of Staphylococcus and Pseudomonas, and little or no *Pasteurella*. Unweighted UniFrac does not differentiate between high and low abundance. Weighted UniFrac does, placing the infected sample in the bottom right corner of that plot. Information UniFrac weights everything in the infected sample close to zero, as taxa are present in either very high or very low abundance, while weighting Staphylococcus and Pseudomonas in the other samples highly (around 0.4) due to their 15-20% abundance. Ratio UniFrac recognizes that the infected sample has a taxonomic abundance very far from the geometric mean abundance. For these reasons information and ratio UniFrac are more adept at picking up outliers with uneven distributions, even if the taxa are shared by other samples.

### 12 Monoculture data

**Fig 8:**
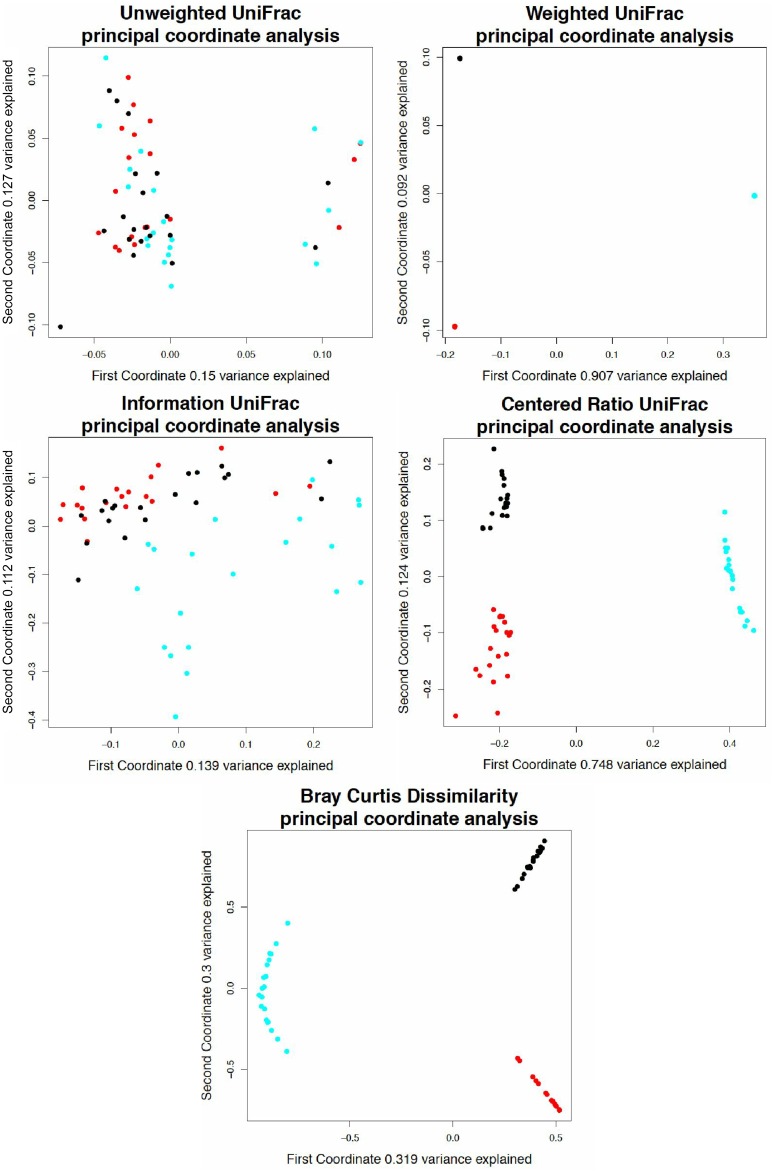
Analysis of simulated monocultures using different UniFrac weightings. A principal coordinate analysis of a simulated 16S rRNA gene tag experiment based on the breast milk data. Red samples are dominated at 97% by *Pasteurella*, black samples are dominated by *Pseudomonas*, and cyan samples are dominated by *Staphylococcus*. Note that while information Unifrac appears to separate the samples reasonably well visually, the amount of variance explained by the first two coordinates is much lower than even weighted UniFrac.

Fig 8 shows the results of a simulated near monoculture dataset that demonstrates how each metric behaves with extreme data. Each sample in the monoculture dataset is 97% dominated by one of three taxa. However, within the remaining 3% there is variation and sparsity in the counts.

Unweighted UniFrac, being a binary test, detects only the variation in the remaining 3% of counts, without showing the difference in the near monocultures. Weighted UniFrac detects only the difference in the identity of the monoculture, and the separation is driven by phylogenetic distance—the pairwise distance from *Pasteurella* to *Staphylococcus* and *Pseudomonas* to *Staphylococcus* is just over 0.9 on the phylogenetic tree while the distance from *Pasteurella* to *Pseudomonas* is 0.45. This is in correspondence with the PCoA plot where the first coordinate (which separates the *Staphylococcus* species from the other two) explains over 90% of the variance in the data set.

Information UniFrac is known to not perform very well for monocultures, due to taxa with very high and low proportional abundances having uncertainty information values close to zero (Fig 5). While the samples separate visually with information UniFrac, the variance explained by the separation is low, and the distance matrix does not separate the three groups by hierarchical clustering. Ratio UniFrac and Bray Curtis both separate the samples by monoculture, and also differentiate the samples by their minor variations, showcasing a more representative perspective of this data set.

If the samples are hierarchically clustered, the three groups separate perfectly with weighted UniFrac, ratio UniFrac, and Bray Curtis dissimilarity, but not with unweighted UniFrac or information UniFrac.

## Discussion

As shown in the tongue and buccal mucosa data set, unweighted UniFrac is perfectly sufficient for data sets with a notable difference. However, in data sets with no difference or a very small difference between groups such the uniform tongue dorsum data set, unweighted UniFrac is the least reliable and we found that it may produce wildly different results depending on rarefaction and sequencing depth. This can result in spurious groups, or inclusion of samples in the wrong groups.

We found weighted UniFrac, information UniFrac, ratio UniFrac, and Bray-Curtis methods to be more reliable choices. We suggest that investigators use several methods as they can detect outliers in different circumstances. When an outlier is detected by any metric, an investigation is warranted, as with the example in the breast milk data set.

We do not believe that any of these weightings are a perfect model for microbiome data. Each tool is prone to its own set of weaknesses. If the difference in groups is driven by presence/absence then UniFrac is a reasonable choice. If the difference is driven by a linear abundance, then weighted UniFrac is a good choice. Information UniFrac and ratio UniFrac are useful for examining data sets that contain a similar set of taxa between groups. Information and ratio UniFrac are especially useful for examining data sets that have more subtle variations, due to their non linear nature. In any case, inspection should be done to make sure that the tool used accurately represents the data.

In summary, with the addition of information UniFrac and ratio UniFrac, biologists have more tools at their disposal to prevent spurious interpretations, detect outliers, and ultimately understand their data better.

## Supporting Information

S1 Fig Principal Coordinate Analysis derived from GUniFrac distance matrices. GUniFrac was run with an alpha of 0 and 0.25. Note that GUniFrac, like QIIME, prunes the tree with every pairwise comparison. That is, the phylogenetic tree used for the distance calculation for each pair of samples can be different. The resulting measurements are a dissimilarity, not a distance. Additionally, QIIME gives slightly different values from GUniFrac, but the source of this (likely an additional normalization) is not known.
(TIFF)

S2 Fig Principal Coordinate Analysis derived from GUniFrac distance matrices. GUniFrac was run with an alpha of 0.5 and 0.75. Note that GUniFrac, like QIIME, prunes the tree with every pairwise comparison. That is, the phylogenetic tree used for the distance calculation for each pair of samples can be different. The resulting measurements are a dissimilarity, not a distance. Additionally, QIIME gives slightly different values from GUniFrac, but the source of this (likely an additional normalization) is not known.
(TIFF)

S3 Fig Principal Coordinate Analysis derived from GUniFrac distance matrices. GUniFrac was run with an alpha of 1 (equivalent to weighted UniFrac), plus unweighted UniFrac for comparison. Note that GUniFrac, like QIIME, prunes the tree with every pairwise comparison. That is, the phylogenetic tree used for the distance calculation for each pair of samples can be different. The resulting measurements are a dissimilarity, not a distance. Additionally, QIIME gives slightly different values from GUniFrac, but the source of this (likely an additional normalization) is not known.
(TIFF)

S4 FigWeighted UniFrac is a dissimilarity with tree pruning.Here, the weighted UniFrac measurements without tree pruning are: $W_{AB}=\frac{121}{252}$, $W_{BC}=\frac{111}{252}$, and $W_{AC}=\frac{11}{12}$. With tree pruning, the measurements are: $W_{AB}=\frac{121}{252}$, $W_{BC}=\frac{111}{252}$, and *W*_*AC*_ = 1, which fails the triangle inequality.(TIFF)
