# Supplementary figures and images for: Expanding the UniFrac Toolbox
Source: PLoS One. 2016 Sep 15;11(9):e0161196. doi: 10.1371/journal.pone.0161196 (PMC5025018; doi:10.1371/journal.pone.0161196)

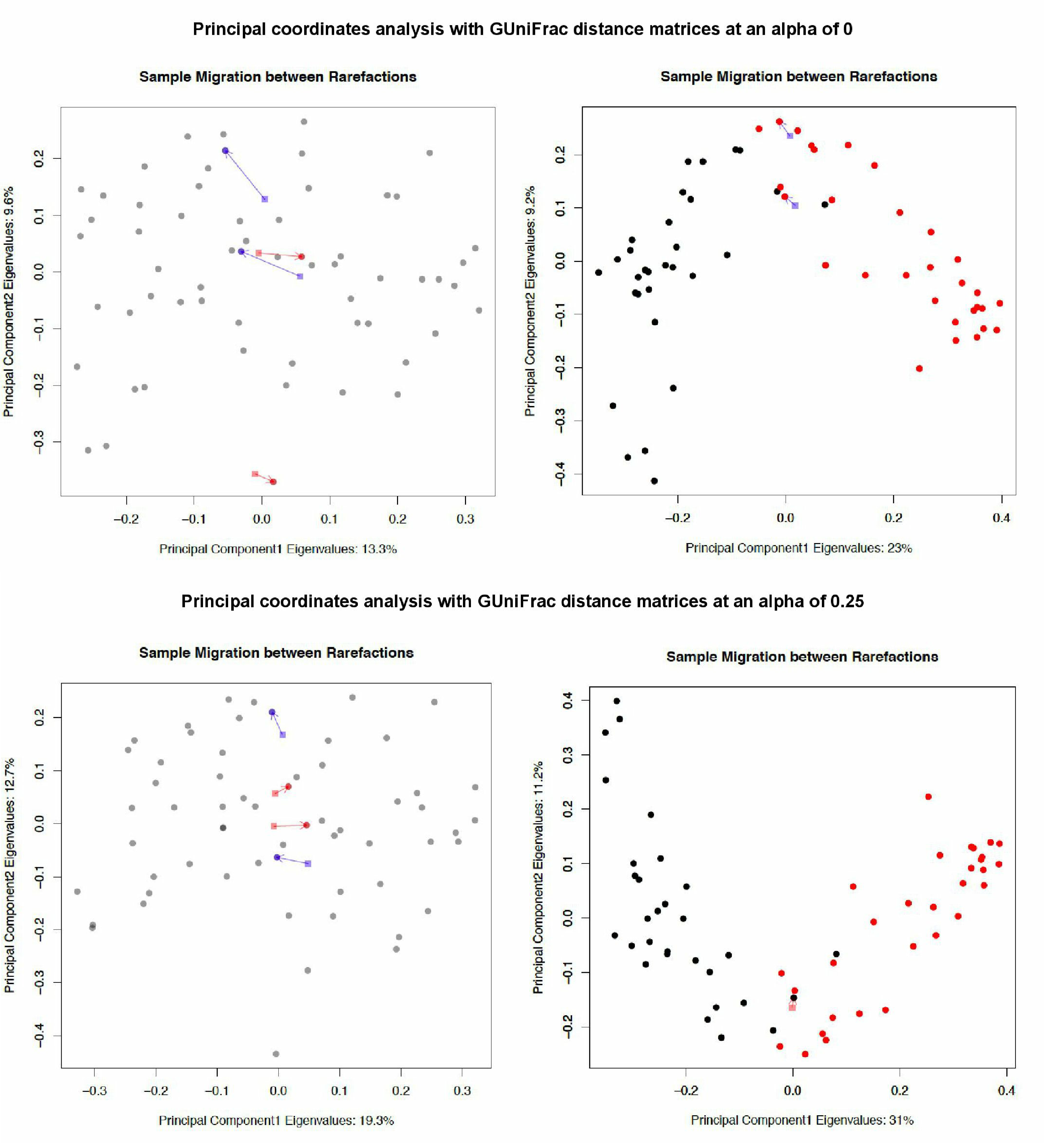

Supplement: S1 Fig — GUniFrac was run with an alpha of 0 and 0.25. Note that GUniFrac, like QIIME, prunes the tree with every pairwise comparison. That is, the phylogenetic tree used for the distance calculation for each pair of samples can be different. The resulting measurements are a dissimilarity, not a distance. Additionally, QIIME gives slightly different values from GUniFrac, but the source of this (likely an additional normalization) is not known. (TIFF) [file pone.0161196.s001.tiff]

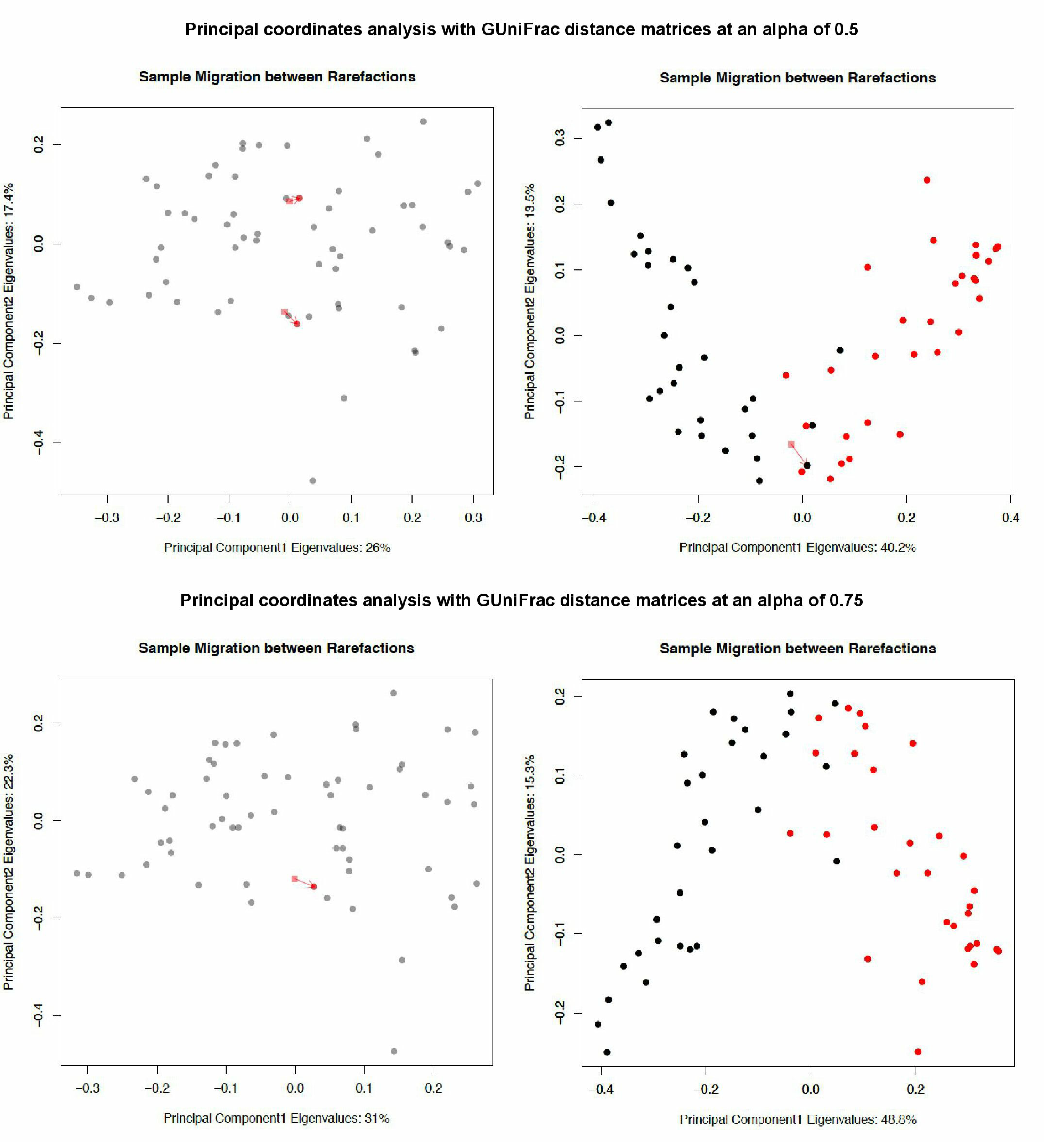

Supplement: S2 Fig — GUniFrac was run with an alpha of 0.5 and 0.75. Note that GUniFrac, like QIIME, prunes the tree with every pairwise comparison. That is, the phylogenetic tree used for the distance calculation for each pair of samples can be different. The resulting measurements are a dissimilarity, not a distance. Additionally, QIIME gives slightly different values from GUniFrac, but the source of this (likely an additional normalization) is not known. (TIFF) [file pone.0161196.s002.tiff]

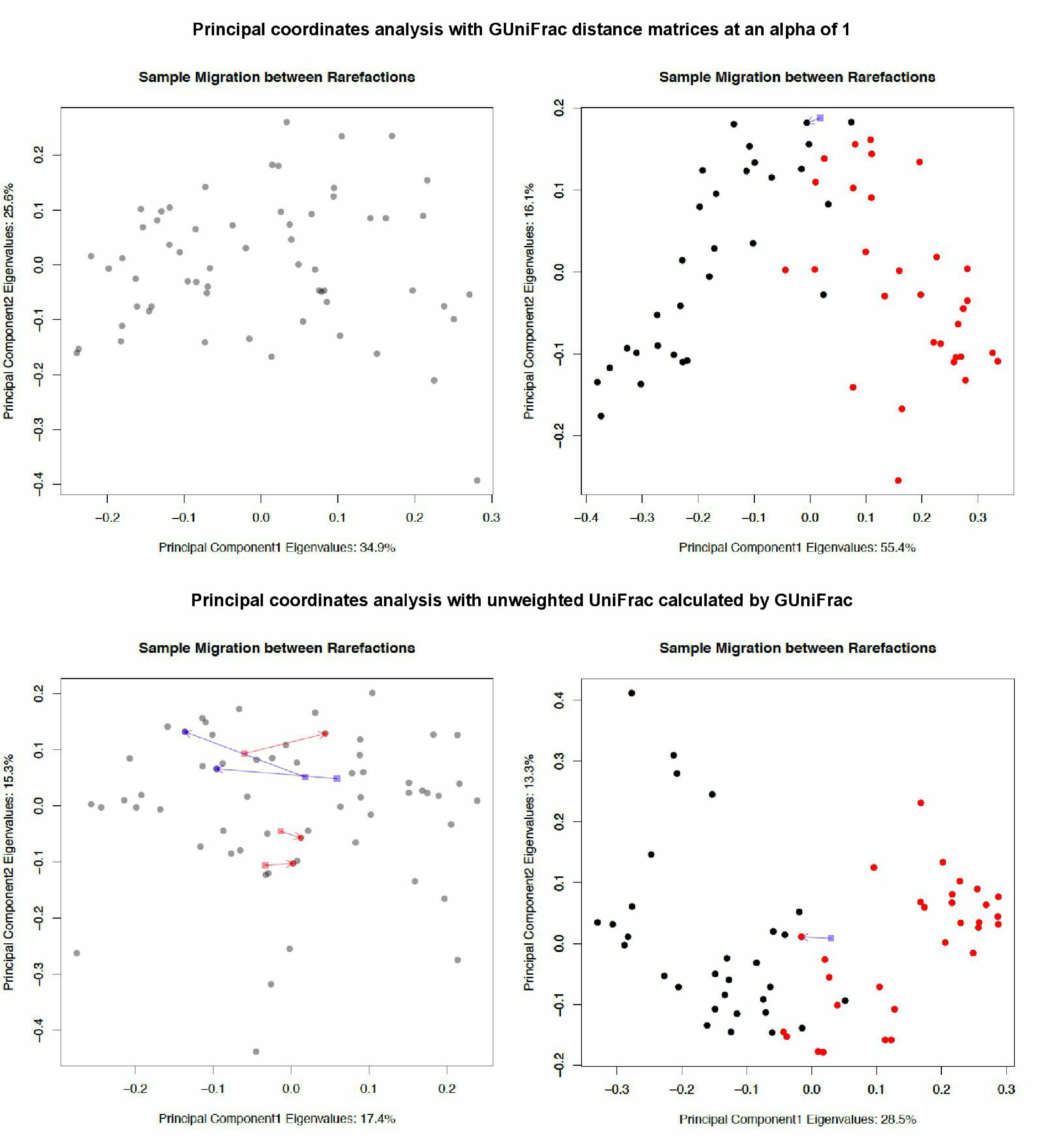

Supplement: S3 Fig — GUniFrac was run with an alpha of 1 (equivalent to weighted UniFrac), plus unweighted UniFrac for comparison. Note that GUniFrac, like QIIME, prunes the tree with every pairwise comparison. That is, the phylogenetic tree used for the distance calculation for each pair of samples can be different. The resulting measurements are a dissimilarity, not a distance. Additionally, QIIME gives slightly different values from GUniFrac, but the source of this (likely an additional normalization) is not known. (TIFF) [file pone.0161196.s003.tiff]

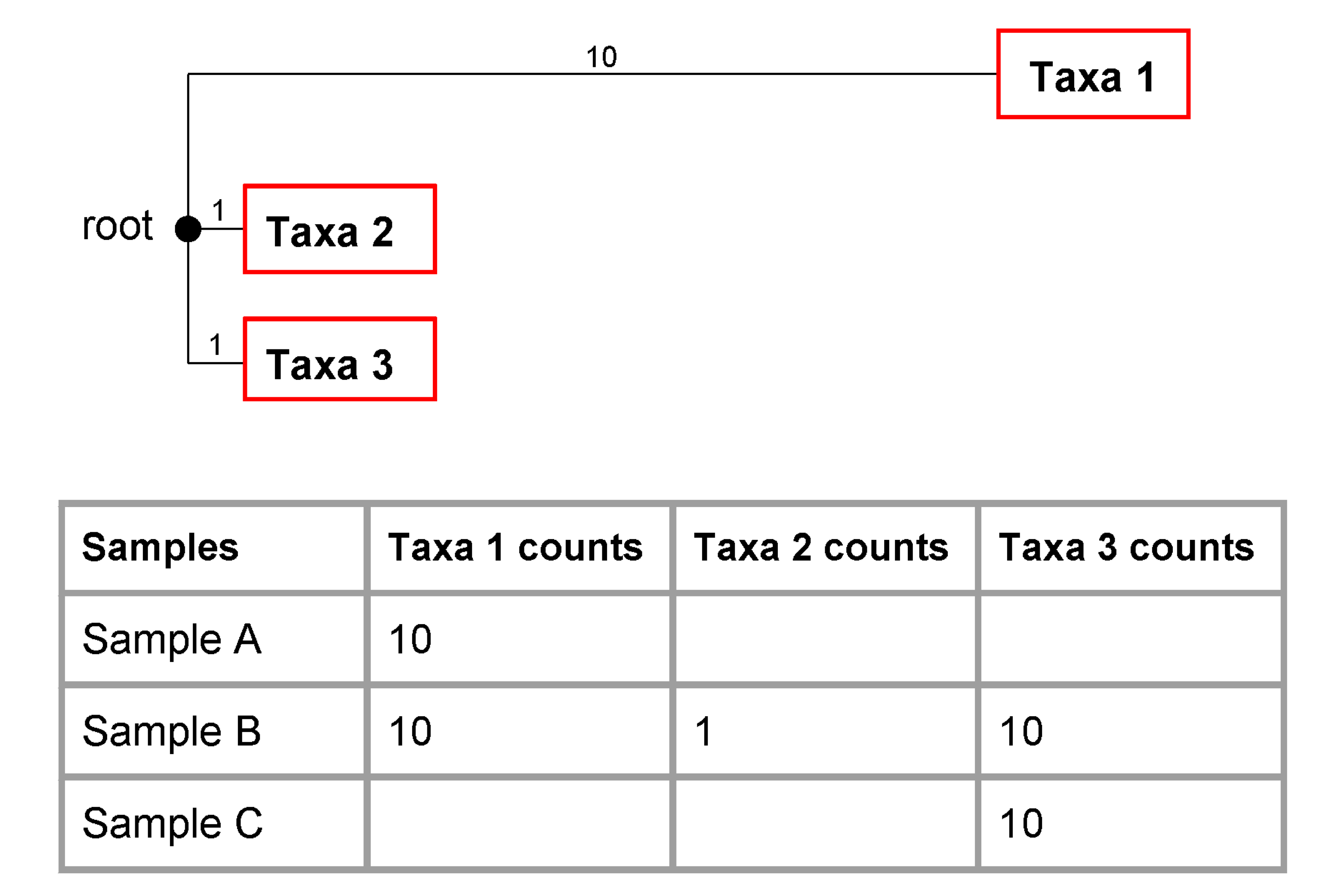

Supplement: S4 Fig — Here, the weighted UniFrac measurements without tree pruning are: WAB=121252, WBC=111252, and WAC=1112. With tree pruning, the measurements are: WAB=121252, WBC=111252, and WAC = 1, which fails the triangle inequality. (TIFF) [file pone.0161196.s004.tiff]
